# Towards Stochastic Optimization-Based Electric Vehicle Penetration in a Novel Archipelago Microgrid

**DOI:** 10.3390/s16060907

**Published:** 2016-06-17

**Authors:** Qingyu Yang, Dou An, Wei Yu, Zhengan Tan, Xinyu Yang

**Affiliations:** 1 SKLMSE Lab, School of Electronic & Information Engineering, Xi’an Jiaotong University, Xi’an 710049, China; 2 School of Electronic & Information Engineering, Xi’an Jiaotong University, Xi’an 710049, China; adkaka.an@gmail.com (D.A.); hh621823@stu.xjtu.edu.cn (Z.T.); yxyphd@mail.xjtu.edu.cn (X.Y.); 3 Department of Computer and Information Sciences, Towson University, Towson, MD 21252, USA

**Keywords:** archipelago microgrid, electric vehicles (EVs), two-stage stochastic programming, scheduling

## Abstract

Due to the advantage of avoiding upstream disturbance and voltage fluctuation from a power transmission system, Islanded Micro-Grids (IMG) have attracted much attention. In this paper, we first propose a novel self-sufficient Cyber-Physical System (CPS) supported by Internet of Things (IoT) techniques, namely “archipelago micro-grid (MG)”, which integrates the power grid and sensor networks to make the grid operation effective and is comprised of multiple MGs while disconnected with the utility grid. The Electric Vehicles (EVs) are used to replace a portion of Conventional Vehicles (CVs) to reduce CO${}_{2}$ emission and operation cost. Nonetheless, the intermittent nature and uncertainty of Renewable Energy Sources (RESs) remain a challenging issue in managing energy resources in the system. To address these issues, we formalize the optimal EV penetration problem as a two-stage Stochastic Optimal Penetration (SOP) model, which aims to minimize the emission and operation cost in the system. Uncertainties coming from RESs (e.g., wind, solar, and load demand) are considered in the stochastic model and random parameters to represent those uncertainties are captured by the Monte Carlo-based method. To enable the reasonable deployment of EVs in each MGs, we develop two scheduling schemes, namely Unlimited Coordinated Scheme (UCS) and Limited Coordinated Scheme (LCS), respectively. An extensive simulation study based on a modified 9 bus system with three MGs has been carried out to show the effectiveness of our proposed schemes. The evaluation data indicates that our proposed strategy can reduce both the environmental pollution created by CO${}_{2}$ emissions and operation costs in UCS and LCS.

## 1. Introduction

With the development of the smart grid, microgrids (MGs) are becoming miniaturized, independent and community-based continuously. As a typical energy-based Cyber-Physical System (CPS) supported by Internet of Things (IoT) techniques [1], MGs integrate both physical elements in the power grid and cyber elements (sensor networks, communication networks, and computation core) to make the power grid operation effective. Such a complex system calls for novel approaches for system design and modeling and new techniques to manage energy resource efficiently.

Due to highly intermittent and uncertainties from both physical power grid and cyber network components, new techniques for MG modeling and management should be developed to enhance energy efficiency [2]. Specifically, the emerged Islanded Micro-Grids (IMG) [3] have received growing attention due to the self-balance and stability under voltage fluctuations. The switch in IMG connected to the utility grid can turn off the connection, in case the malfunction resulted from the main grid, including upstream disturbances, voltage fluctuation, *etc.* [4]. The operation of IMG is able to effectively mitigate the detrimental situations raised by the utility grid, reduce the operation cost and enhance the reliability when a power shortage occurs.

Due to flexibility of charging and discharging Electric Vehicles (EVs) in IMG, energy efficiency and environmental friendship, EVs, as a key component in IMG, have drawn much research attention in recent years. With the integration of EVs in IMG, effective scheduling schemes are required to help smooth the load curve and reduce the environmental emissions. To this end, a large number of research efforts have been conducted on EVs. For example, an objective of minimizing the operation cost and maximizing the profit of IMG was studied by scheduling the EV charging and battery swapping station with a fuzzy control approach in [5]. Saber and Venayagamoorthy in [6] investigated the overall scale of EVs under the objective of reducing the CO${}_{2}$ emission and operation cost. Nonetheless, their proposed scheme only used EVs to replace all Conventional Vehicles (CVs) while the local unit capacity and the investment costs were not considered.

In an IMG, the installed local units’ generation need to meet the load demand during the peak time duration, combining with the generation of Renewable energy sources (RESs). In comparison with the MGs connected to the utility grid, a larger capacity should be required in an IMG, which leads to an expensive investment cost. In addition, if there are sufficient RESs in an IMG, the surplus energy will be wasted after satisfying the load demand and filling up the storage device, leading to an inefficient energy usage. To this end, as an essential load and energy storage device, EVs offer great flexibility to influence the energy efficiency, stability, and environment benefits of IMG significantly. Thus, it is critical to develop effective scheduling schemes for EVs in an IMG.

Nonetheless, the intermittent and randomness of RESs (e.g., wind and solar) will create great challenges to the stability and reliability of IMG operation [7]. In particular, the forecast accuracy of the uncertain parameters are essential to the energy efficiency. For instance, a lower prediction accuracy will lead to more reverse requirements to compensate the errors in the case that the forecast generation is more than the actual generation. In addition, the precise prediction of parameters (electricity prices, *etc.*) helps the consumers make rational decisions of EV charging and discharging, thus avoiding generating new peak load for the system. Therefore, how to tackle the uncertainties in an IMG is a critical issue.

To address these issues, in this paper, we first propose a novel IMG system, named “archipelago micro-grid (IMG)”, which is disconnected from the utility grid and composed of several connected MGs. In such a system, the energy demanded is fully satisfied by distributed energy generations. In the proposed IMG, we formalize the optimal scheduling problem, which aims to minimize the emission and operation cost by arranging the rational charging/discharging plan and deploying a reasonable number of EVs while considering various uncertainties.

The key contributions of this paper can be summarized as follows:

- To improve the energy efficiency and ensure the stability of IMG, we propose a novel multi-microgrid system concept named “archipelago microgrid”. In our proposed system, the required power will be fully provided by the distributed power generation in local MGs. Through the energy transmission controlled by a MicroGrid Center Controller (MGCC, aggregator) among the MGs, the emission and operation cost created by local units can be mitigated. The proposed system is helpful in reducing operation cost and enhancing the stability of the grid.
- We propose a Stochastic Optimal Penetration (SOP) model, aiming to minimize the cost of the proposed “archipelago microgrid” system. To address the uncertainties from RESs and electricity prices, the optimization problem is formalized as a two-stage stochastic programming problem. In the formalized problem, the uncertain parameters such as wind, solar generation capacity are captured by the Monte Carlo-based method [8]. For the sake of comparison, the Deterministic Optimal Penetration (DOP) model based on our prior work [9] is introduced as a baseline scheme. In the optimization problem, the emission cost created by CVs and local units, the operation cost of startup/shutdown expense of units, tariff compensation, battery capacity degradation, and power losses are considered in the optimization model. The proposed stochastic model offers a desired flexibility between the environmental and economic benefits by seeking the optimal number of EVs with the considerations of the RESs and units generation limits.
- To achieve the minimization of emission and operation cost, we propose the following two schemes to schedule the optimized scale of EVs: (i) Unlimited Coordinated Scheme (UCS); and (ii) Limited Coordinated Scheme (LCS). In UCS, we consider that all the surplus energy is utilized to charge as many EVs as possible. In this ideal scenario, the mutual transmission among MGs is also allowed to avoid energy wasted. Nonetheless, peak load limits and residence preference of energy usage should be considered in practice. To this end, we propose the LCS to achieve a more realistic number of penetrated EVs to minimize the total cost.
- We carry out an extensive simulation study on a modified IEEE 9-bus system to demonstrate the effectiveness of the proposed SOP model using the two scheduling schemes. The simulation results shows that, after addressing uncertainties, the emissions and operation cost are reduced in comparison with the deterministic-based optimization model. In addition, with respect to the two schedule schemes in SOP, the LCS has been proven to be more effective on arranging the scale of EVs and realizing the emission and operation cost reduction than the UCS. Our experimental data shows that, in comparison with a baseline non-coordinated average scheme, 15.2% of emissions can be reduced and 11.2% cost can be saved in the LCS, we conduct sensitivity analysis to validate the impact of different parameters on the optimal solution.

Part of this work was published in [9]. Based on the much shorter conference version, we have made substantial extensions in this journal submission. The rest of the paper is organized as follows, we present system models in Section 2. In Section 3, we show the formalization of the SOP problem and propose our scheduling schemes. In Section 4, we give the performance evaluation results to demonstrate the effectiveness of our proposed schemes. We present the literature review in Section 5, and we conclude the paper in Section 6, respectively.

## 2. System Models

In this section, we first present the system model of our archipelago microgrid and then describe the market model used in the paper.

### 2.1. Archipelago Microgrid Model

**Figure 1:**
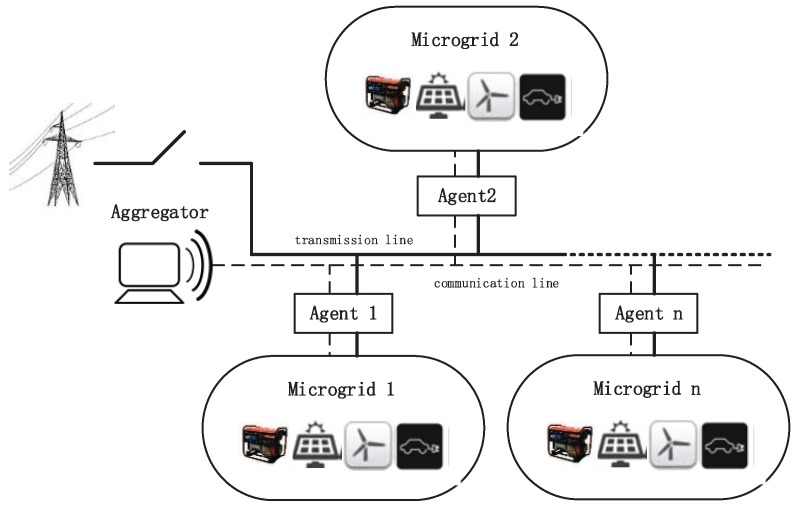
Structure of the archipelago microgrid.

In this paper, in order to refrain from the voltage fluctuations and upstream disturbances caused by the utility grid, while increasing the reliability of local MGs, we propose the concept of an independent and self-sufficient system, called “archipelago microgrid”. The basic structure of “archipelago microgrid” is illustrated in Figure 1. As shown in the figure, the system is a typical energy-based Cyber-Physical System supported by Internet of Things (IoT) techniques, which consists of both cyber and physical components.

The physical components are comprised of local MGs, the interconnection transformer which ensures the power transmission among local MGs. In each MG, the diesel unit and various RES generation devices are used to produce the required energy. The installed capacity of all distributed generators will meet the load demand of each MG in order to avoid the occurrence of power outage. In addition, RES generators, which consist of solar panels and wind turbines, are integrated in each MG. The loads in MGs are divided into two types: EVs and non-EV loads. Through G2V (Grid-to-Vehicle) and V2G (Vehicle-to-Grid) technologies, the EVs can be managed to charge or discharge at different time slots according to the current power consumption, while the non-EV loads are unable to schedule.

In addition, the cyber components guarantees the information transmission in the system, which are typical sensor networks composed of Advanced Metering Infrastructure (AMI)—in which, the data measured by sensors, including timely energy consumption, load forecasting and RES related information within the MG, will be collected by the MG agents and transmitted to the MGCC. The entire region will be monitored by the system MGCC that can send or receive various information from or to the agents supported by AMI (sensor networks).

### 2.2. Market Model and Aggregator

In order to penetrate the reasonable number of EVs while minimizing the operation and emission cost (according to the timely supply and demand information), we consider that when there exists energy surplus in some MGs, in order to reduce the CO${}_{2}$ emission operation cost, we can develop an effective power scheduling scheme for the local unit and arrange the most charged EVs to fully utilize the distributed power generation. Then, the remaining power can be delivered to other MGs for the purpose of load offset and EV charging. In addition, we need to discharge EVs when the power shortage occurs. The discharged power can not only address the need of supply, but also reduce substantial generation cost.

As the “central nervous” of the whole system, the responsibility of the MGCC (MicroGrid Center Controller) is to minimize the CO${}_{2}$ emission and operation cost. We assume that all MGs are have the same interests and the surplus power transport among them is free. Nonetheless, because of the transmission voltage and resistance of the line between the MGs, power losses are unavoidable when transmitting power in MV (Motor Vehicle) networks [10]. Obviously, the aggregator is able to preferentially allocate the transported power between the MGs with less power losses. According to the collected information associated with the electrical quantity and RES generation from MGs, the MGCC can compute the optimized scheduling plan for the EVs and deploy a number of EVs for charging.

When the RES generation in some MGs is not enough for the load demand (including EVs), they will first send a request signal to the MGCC to ask for help. If there is any surplus energy provided by other MGs, the MGCC will dispatch the remaining power to the requester automatically to meet the load demand and EV charging activities. If there is no remaining energy left, the MGCC will first exploit the fully charged EV to discharge without affecting its normal operation, and then, start up the local unit to meet the power shortage. We consider EVs as the storage device in the system, thus the unused energy after scheduling will not affect the schedule in the next time slot. In addition, we assume that EVs only charge and discharge once a day. Therefore, the scheduling at each period will be independent, meaning that the scheduling results at time slot *t* will not affect the result at any other time slot. Once the schedule is complete, the historical data will be stored. The MGCC will monitor the operation of the system again and prepare for the scheduling at the next time slot.

## 3. Our Approach

In this section, we first present the basic idea of our approach. We then give the formulated optimization model and introduce the proposed scheduling schemes. Table 1 show all key notations used in the paper.

### 3.1. Basic Idea

To improve the energy efficiency and reduce the cost of IMG, in this paper, we propose a novel system, named “archipelago microgrid” structure. The proposed system has the ability of enabling the interaction among MGs and isolating them from the utility gird, as well as the constant and low electricity price of transported power among local MGs.

Notice that the diesel generators used in the archipelago microgrid will lead to lots of CO${}_{2}$ emissions. The existence of CVs are a source of pollution as well. With more attention to environmental issues, emission cost has become an essential aspect in MGs. In addition, the startup/shutdown associated cost, the life loss of lithium batteries, and the amount of fuel utilized for power generation lead to a greater operation cost. Thus, it is required for an optimized penetration of EVs to protect the environment and realize a great reduction on CO${}_{2}$ emission. In addition, as the storage device is in the archipelago microgrid, we can make a better response to the timely load levels by regulating the number of EVs to be charged at low demand intervals or discharged during the power shortage intervals. By doing so, a reduction of emissions and generation cost can be achieved. Nonetheless, an increase of other costs such as battery capacity degradation and power losses will be introduced. To this end, an optimization problem aims to minimize the emission and operation cost associated with the scale (number) of EVs is proposed in the paper. The objective contains two aspects of costs: emission and operation. In order to increase the flexibility in the scheduling process, we set the different weighted ratios for the environmental and economic benefits. The larger weighted factor of emission cost set, the more attention is focused on environmental issues, and *vice versa*.

Nonetheless, variabilities and uncertainties raised by RESs make the energy resource management in the system with MGs remain a challenging issue. On the supply side, unlike the traditional energy resources, wind and solar power output are highly uncertain and unpredictable. Even a small error in the prediction can result in great errors in real-time operations. On the demand side, factors (natural disasters, plug-in vehicles, personal habits of using energy, weather and temperature, *etc.*) make it difficult to accurately predict the usage of energy. The effectiveness of integrating MGs can be affected by those uncertainties, including the local generator output, reverse requirement, *etc*. To this end, in this paper, we formalize the optimal EV penetration problem as a two-stage stochastic programming problem. In the formalized problem, we first consider the parameters that could capture different uncertainties, including the randomness of renewable energy resource prediction such as wind and PV. We then leverage the Monte Carlo-based method to construct scenarios according to the distribution functions of those parameters to capture uncertainties.

To minimize the expected cost, we propose two schemes to arrange the number of EV charging or discharging, which are denoted as Unlimited Coordinated Scheme (UCS) and Limited Coordinated Scheme (LCS), respectively. In our schemes, the surplus energy is allowed to be transported among the local MGs and the power can be used for charging the EVs and offsetting the non-EV load. Particularly, in the UCS, all the surplus energy is allowed to charge as many EVs as possible, while tariff compensation for the residential users who cannot charge EVs in their preference intervals and the system peak load limits are considered in LCS.

### 3.2. Problem Formulation

We now formalize the optimal emission and operation cost problem as a two-stage stochastic programming problem. We first present the objective function and then introduce the constraints. Generally speaking, the stochastic programming process is a method that aims to minimize the cost in a number of scenarios constructed by Monte Carlo-based method, while being obligated to uncertainties in the problem. The basic idea of the two-stage programming process is to conduct a recursion process to make a corrective decision after the occurrence of random events.

In our two-stage stochastic process, the inputs to the underlying problem include the scenarios generated by the Monte Carlo-based method, which represent the uncertainties in renewable energy outputs, including wind and solar, as well as electricity prices. The outputs of the optimization problem are composed of the first-stage decisions and the second-stage decisions. The first-stage decisions include the commitment statuses of all conventional units, power dispatch of all generation units, and losses created by the power transmission among MGs. The second-stage decisions consist of EV charging decisions and reverse to compensate the prediction errors when the actual RES generation is lower than the predicted amount. In addition, for the purpose of comparison, a Deterministic Optimal Penetration (DOP) model is considered as a baseline scheme, in which the parameters such as renewable energy generation are based on the persist forecast, and the electricity prices are able to be predicted by techniques (e.g., Auto-Regressive and Moving Average (ARMA) [11] and Support Vector Machines (SVM) [12]).

#### 3.2.1. Stochastic Optimal Penetration (SOP) Model

**SOP modeling:**

In order to minimize the emission and operation cost with considering the various uncertainties, the objective function of the Stochastic Optimal Penetration (SOP) model is as follows:

(1) $$\begin{array}{c} \min\sum_{i=1}^{NM}\sum_{t=1}^{NT}\left\{w\left[\sum_{j=1}^{NG}E\left(P_{i,j,t}\right)+E_{CV}\left(N_{CV,i}\right)\right]\right.+\left(1-w\right)\\ \left[\sum_{j=1}^{NG}\left(SU_{i,j}+SD_{i,j}\right)\right.+\sum_{j=1}^{NG}Cost\left(P_{i,j,t}\right)+\sum_{s=1}^{NS}\rho_{s}\sum_{r=1}^{NR}E(P_{i,r,t})\\ +\lambda_{1}\left(\eta^{cha}\sum_{k=1}^{n_{i,t}^{cha}}P_{EV,i,t,k}^{cha}I_{i,t}+\frac{\sum_{k=1}^{n_{i,t}^{dis}}P_{EV,i,t,k}^{dis}U_{i,t}}{\eta^{dis}}\right)\\ \left.\left.+\lambda_{2}P_{i,t}^{loss}+\sum_{s=1}^{NS}\rho_{s}\mu \sum_{k=1}^{n_{i,t}}P_{EV,i,t,k}^{cha}\left(r_{t}-r_{\min}\right)\right]\right\} \end{array}$$

We now explain the SOP model in detail. The emission cost consists of the local unit emission and CVs emission, respectively. The startup/shutdown and generation cost of local units, the penalty of battery capacity degradation, the power loss created by transmission, and tariff compensation are the examples of the operation cost according to Equation (1).

The detail of the objective function is that, the first part $E\left(P_{i,j,t}\right)$ and second part $E_{CV}\left(N_{CV,i}\right)$ in our model capture the emission costs caused by the local units and CVs, respectively. The CO${}_{2}$ emission of the local units depends on the consumption of fuel, and the CV emissions are directly based on their number and traveling distance. Because the PV and wind are clean energy resources, there will be no pollution caused during the generation of such resources.

The third part $SU_{i,j}+SD_{i,j}$ and fourth part $Cost\left(P_{i,j,t}\right)$ in the SOP model represent the startup/shutdown and operation cost of local units, respectively. In our MG model, the distributed generation is composed of diesel generators, PV panels and wind turbines as shown in Figure 1. Notice that as the installation cost for PV panels and wind turbines integration is associated with long-term issues, we only consider the operation cost of diesel generators in the paper.

In the fifth part, $E(P_{i,r,t})$ is the cost associated with utilizing reverses to compensate the errors when the prediction output is larger than the actual RES generation. We define $\rho_{s}$ as the probability of scenario *s* and denote NS as the number of scenarios.

The sixth part includes the equation on the third line of the Equation (1), which represents the penalty of lithium battery degradation in EVs. The seventh part $\lambda_{2}P_{i,t}^{loss}$ presents the penalty of the power loss raised by the power transmission among MGs. Due to the line resistance, power loss is inevitable while the surplus is transported from one MG to another. To alleviate the power transmission loss, a Medium Voltage (MV) between two grids is used. According to [10,13], the power transmission losses can be obtained through

(2) $$P_{loss}=\frac{R_{i,l}Q_{i,l,t}^{2}}{V^{2}}$$

where $R_{i,l}$ is the line resistance between MG *i* and *l*, and *V* indicates the transfer voltage between the interconnected MGs. In order to facilitate the management of the system, all transfer voltages between different MGs are considered to be the same value.

The eighth part, which is located after the last plus sign, represents the tariff compensation, enabling a realistic consideration of residents’ behaviors. In real-world practice, all the consumers tend to charge EVs at a lower electricity price interval. Nonetheless, due to the limitation of load and generation capacities, it is impossible to depose all the EVs in the same time slot. To this end, the aggregator can defer a portion of EVs to other time slots with more RESs and low loads. Notice that this behavior violates the consumer’s aspiration and incurs an extra cost since the charging occurs at high price slots. To this end, it is essential to introduce tariffs to make up for the residential users’ additional overhead raised by not being charged at their desired slots. In particular, with the increase of *μ*, more compensation can be paid and the number of EVs can be significantly reduced too.

**SOP constraints:**

In the optimization problem, the constraints need to be considered as well. The first constraint is to balance power. For each MG in the system, the following power balance constraint needs to be satisfied:

The first constraint is to balance the power. For each MG in the system, the following power balance constraint needs to be satisfied:

(3) $$\begin{array}{c} P_{load,i,t}+\sum_{l\neq i}Q_{i,l,t}+\sum_{k=1}^{n_{i,t}}P_{EV,i,t,k}^{cha}\times I_{i,t}=\sum_{j=1}^{NG}P_{i,j,t}+\\ P_{i,r,t}+P_{Pv,i,t}+P_{Wind,i,t}+\sum_{l\neq i}Q_{l,i,t}-\sum_{l\neq i}Q_{loss,l,i,t}\\ +\sum_{k=1}^{n_{i,t}}P_{EV,i,t,k}^{dis}I_{i,t}\forall i,t \end{array}$$

where the left parts in the equality indicate the the local units generations, RES generations (e.g., solar and wind), the discharged power from EVs, and the transported power from other MGs, the right parts present the amount of demand, which consists of non-EV loads, the amount of power charged to the EVs, power losses resulted from line resistance, and the amount of the surplus power transmitted to other MGs.

Second, the local units constraints should be satisfied. Recall that the fuel consumption features of diesel generators are varied due to different levels of power rated. In general, the fuel used for the unit power generation will reduce as the increase of the rated level of power used by generators. To describe the characteristic of diesel generators, we use a linear model for the fuel consumption that considers different types of generations by [14]:

(4) $$FC_{i,j}=a_{i,j}P_{i,j,t}+b_{i,j}P_{rated,i,j},\forall i,j,t$$

where $FC_{i,j}$ is the fuel consumption of diesel generator *j* in MG *i*. For the operation cost of diesel generators, we use the cost model developed by [15] as follows:

(5) $$Cost\left(P_{i,j,t}\right)=\;\alpha_{i,j}x_{i,j,t}+\beta_{i,j}P_{i,j,t},\;\forall i,j,t\;$$

where the Cost(.) represents the operation cost of diesel generator *j* in MG *i*. In addition, the unit operation constraints can be expressed as:

(6) $$\begin{array}{c} P_{i,j}^{\min}x_{i,j,t}\leq P_{i,t,j}\leq P_{i,j}^{\max}x_{i,j,t}\\ P_{i,j,t}-P_{i,j,t-1}\leq Ru_{i,j}\left(1-y_{i,j,t}\right)+P_{i,j}^{\min}y_{i,j,t}\\ P_{i,j,t-1}-P_{i,j,t}\leq R(d_{i,j}\left(1-z_{i,j,t}\right)+P_{i,j}^{\min}z_{i,j,t}\\ y_{i,j,t}+z_{i,j,t}\leq 1,y_{i,j,t}-z_{i,j,t}=x_{i,j,t}-x_{i,j,t-1} \end{array}$$

where the first constraint indicates the power limit of the diesel generator, the second and third constraints show the ramp-up/down limits, and the fourth constraint indicates the relationship among the different operation statuses of generators.

In addition, notice that the unlimited integration of EVs is unrealistic. Thus, the scale of EVs should be constrained. The number of vehicles, including CVs and EVs, can be estimated by the analysis based on the number of residents in each MG. We leverage the model developed in [6,16] to conduct the estimation. Then, we have

(7) $$N_{R}=\frac{R_{V2G}Q_{REC}D_{Total}}{D_{\mathrm{Average}}}$$

where $D_{total}$ indicates the total load demand in each MG per day. $D_{Average}$ is the average daily electricity consumption of residents, which is equal to 2.0833 kW according the results in [6]. $Q_{REC}$ is the vehicle ownership rate of residents, and $R_{V2G}$ is the proportion of residents who participate in the V2G plan. Apparently, with the popularity of the vehicles, it is reasonable to assume that $Q_{REC}=1$. In this paper, we consider that all the residents use V2G technology so that $R_{V2G}=100\%$. Thus, the relationship between vehicle scale and the number of residents is derived as follows:

(8) $$\begin{array}{c} N_{EV,i}=\sum_{t=1}^{NT}n_{i,t}^{cha}\\ N_{V,i}=N_{R,i}=N_{EV,i}+N_{CV,i},\forall i \end{array}$$

where the first equation indicates that the number of EVs is computed by the sum of EVs in each time slot. The second equations means that the number of vehicles is composed of CVs and EVs.

With consideration of load limits, we also assume that the sum of the newly added load caused by EV charging and non-EV load should not be larger than the maximum load capacity ($P_{load,i}^{max}$ ) at each time slot. To this end, the following constraint is proposed to protect the system from generating new load peaks:

(9) $$\sum_{k=1}^{n_{i,t}^{cha}}P_{EV,i,t,k}^{cha}I_{i,t}+P_{load,i,t}\leq P_{load,i}^{\max}$$

where the left parts in the inequality are the consumptions of EVs and non-EV loads, and $P_{load,i}^{\max}$ indicates the limitations of load in MG *i*.

Finally, the charging and discharging constraints of EVs should be considered as well.

(10) $$\begin{array}{c} 0\leq \sum_{t=1}^{NT}P_{EV,i,t,k}^{cha}I_{i,t}\leq P_{EV}^{Max}\\ 0\leq \sum_{t=1}^{NT}P_{EV,i,t,k}^{dis}U_{i,t}\leq \left(1-\phi_{\min}\right)P_{EV}^{Max}\\ \phi_{\min}P_{EV}^{Max}\leq \sum_{t=1}^{NT}\left(\eta^{cha}P_{EV,i,t,k}^{cha}I_{i,t}-\frac{P_{EV,i,t,k}^{dis}U_{i,t}}{\eta^{dis}}\right)\leq P_{EV}^{Max}\\ I_{i,t}+U_{i,t}=1 \end{array}$$

Here, the first two constraints illustrate the charging and discharging limits, and the $\phi_{min}$ refers to the minimum battery energy storage for the regular driving of EVs. The third one guarantees the stored power in EVs. The last constraint is used to avoid the simultaneous operation of charging and discharging at the same time slot.

#### 3.2.2. Deterministic Optimal Penetration (DOP) Model

In comparison with the performance of the SOP model, a Deterministic Optimal Penetration (DOP) model is formulated as a baseline scheme, in which the parameters associated with the uncertainties (ambient temperature, wind speed, *etc.*) are based on a persist prediction and the electricity prices are forecasted by prediction techniques [11]. The constraints from Equations (2) to (10) should also be satisfied. To this end, we introduce the following DOP model as the baseline scheme:

(11) $$\begin{array}{c} \min\sum_{i=1}^{NM}\sum_{t=1}^{NT}\left\{w\left[\sum_{j=1}^{NG}E\left(P_{i,j,t}\right)+E_{CV}\left(N_{CV,i}\right)\right]\right.\\ +\left(1-w\right)\left[\sum_{j=1}^{NG}\left(SU_{i,j}y_{i,j,t}+SD_{i,j}z_{i,j,t}\right)\right.+\sum_{j=1}^{NG}Cost\left(P_{i,j,t}\right)\\ +\lambda_{1}\left(\eta^{cha}\sum_{k=1}^{n_{i,t}^{cha}}P_{EV,i,t,k}^{cha}I_{i,t}+\frac{\sum_{k=1}^{n_{i,t}^{dis}}P_{EV,i,t,k}^{dis}U_{i,t}}{\eta^{dis}}\right)\\ +\sum_{r=1}^{NR}E(P_{i,r,t})+\lambda_{2}P_{loss}+\left.\left.\mu \sum_{k=1}^{n_{i,t}}P_{EV,i,t,k}^{cha}\left(r_{t}-r_{\min}\right)\right]\right\} \end{array}$$

Here, the first and second parts in the DOP model indicate the emission cost raised by the local units and CVs. The third and fourth parts are the startup/shutdown and operation cost of local generators. The fifth and sixth are the penalty of battery degradation of EVs and the power loss created by power transmission among local MGs. The seventh part part is the tariff compensation to make up the overhead of the users for charing EVs at the time slots with higher prices.

Notice that, in comparison with the SOP model, the uncertainties of the renewable energy resources are not considered and addressed. In addition, as essential energy resources in the "archipelago microgrids", the uncertainties raised by RESs could affect the operation of system. Therefore, the DOP model will result in a higher operation cost than the SOP model. Because of this, more traditional energy resources could be utilized to compensate for the prediction errors of the generation from RESs (*i.e.*, the actual generation is smaller than the prediction). To this end, the DOP model is proposed as a baseline scheme to evaluate the effectiveness of the proposed SOP model.

### 3.3. Proposed Scheduling Schemes

We now present the two proposed scheduling schemes to carry out the optimal scale of EVs effectively. To demonstrate the effectiveness of the two schemes, a baseline allocation scheme for EVs, named Uncoordinated Average Scheme (UAS), is also considered. In the following, we describe these three scheduling schemes in detail.

#### 3.3.1. Uncoordinated Average Scheme (UAS)

Since the non-EV loads are inflexible, the average allocation method for EVs is a feasible way to reduce the emission. In the UAS, the surplus of power is used to supply loads preferentially, and then, the remaining power is used to charge EVs. By accumulating all the surplus power in each MG, the maximum allowable number of EVs can be derived. In particular, if there is not enough RES power to meet the average demand of EVs at the time slot *t*, the exceeded EVs will be deferred to the next time slot. By using this scheme, we can plan the maximum number of EVs in each MG to reach the upper limit of vehicle scale. Notice that the UAS is the least desirable scenario since the surplus power is unable to transport among local MGs. It means that all of the excess energy after fulfilling the load and EVs charging will be dissipated. In this scheme, each MG operates as an independent individual without the interconnection with each other, which will directly result in less accommodated EVs and low energy efficiency.

#### 3.3.2. Unlimited Coordinated Scheme (UCS)

To improve the energy efficiency and reduce the emission and operation cost in the system, we propose a new scheme called UCS. In the UCS, the power exchange among MGs is enabled. Specifically, the agent of each MG is able to collect the timely energy usage data (hourly, *etc.*) and inform the MGCC. If any RES energy surplus exists in an MG, the MGCC can assign the excess energy to the insufficient ones for the purpose of minimizing the emission and operation cost. Then, the energy distribution information from the MGCC will be sent to the agents. The agents can dispatch the transported power rationally for non-EV load offsetting and EVs charging. By doing so, the remaining energy in the system can be used and the energy efficiency can be improved. In addition, in this scheme, each MG can utilize all the surplus energy for EV charging. Thus, a larger number of EVs can be accommodated as a result.

#### 3.3.3. Limited Coordinated Scheme (LCS)

Recall that in the UCS, EVs can be deployed without restriction if there is sufficient surplus power existed in the system. Nonetheless, it is unrealistic for the residents to deploy as many EVs as possible at the time slots with the lowest electricity price because of the limited RES generation. In addition, as we mentioned before, the centralized charging is harmful to the stability and reliability of the MGs due to new load peaks shaped.

To overcome this limitation and deploy the practical number of EVs by achieving the desirable objective of the SOP model, we propose a realistic scheduling scheme, denoted as LCS. In the LCS, the agents and MGCC can collect the energy information in real-time with the support of AMI system and dispatch the surplus power to shed the load demand and charge EVs. In particular, regardless of the amount of remaining power, the MGCC will limit the deployed number of EVs, referring to the peak load restrictions, by evaluating the historical peak load data. In this way, a portion of EVs, which can be charged at the time slots with lower prices in the UCS, has to be moved to other slots with higher prices, leading to a more expensive electricity bill for residents. In order to encourage the residents to participate in the scheduling process, a necessary tariff compensation is used in the LCS to make up for the economic losses of residents. Obviously, the LCS considers more realistic scenarios, leading to more convincing results.

To solve the optimization problem in the LCS scheme, we develop an efficient decentralized algorithm to enable the optimal penetration. At the beginning, the Equation (1) is divided into several subproblems according to different scheduling intervals for the sake of a more efficient solution. It is noted that not all the subproblems are valid unless there is surplus power existed in MGs so that we must exclude the invalid ones. Then, we queue the valid subproblems in ascending order of price, so that we can preferentially deal with the optimal EV scale issue in time windows with lower prices. After that, we solve the subproblems and record the optimal EV number $n_{i,k}$ in each MG sequentially, and accumulate the sum of $n_{i,k}$ after each iteration. Obviously, the accumulated sum of EV numbers will increase and approach the upper limit of EV scale in each MG. After a few steps of iteration, if the number of EVs exceeds the upper limit in an MG, the subproblem should be recomputed by considering a new number of EVs as a constraint, which can be expressed to be $n_{i,k}\leq N_{V,i}-\sum_{k-1}n_{i,t}$. In this way, we can ensure that the computed EV number does not exceed the limit of EV scales in each MG. Otherwise, if the number of EVs reaches the upper limit and there is no further energy demand in this MG, the amount of transmitted power will be set to 0 and all of its surplus energy will be assigned to other MGs, in which the EV scales are unsaturated. The iterations will be terminated if the number of EVs reaches the upper limit in each MG. The detailed algorithm is shown in Algorithm 1.

**Algorithm 1** Decentralized Algorithm**Require:**
$n_{i,t}$ is the number of EVs charged at time slot *t* in MG *i***Ensure: $N_{VP}$, $P_{load,i,t}$, $P_{Pv,i,t}$, $P_{Wind,i,t}$**1:q←0;2:Initialization: the scheduling period NT, the estimated vehicles number $N_{V,i}$ in MG *i*. Denote two new constraints (11) and (12) as empty;3:Divide the optimization problem in Equation (1) into subproblems by scheduling intervals and queue the subproblems according to electricity prices ascending.4:Set VP=Group of valid subproblems;5:for t=1:NT6:**if**
$P_{load,i,t}-P_{Pv,i,t}-PWind,i,t\geq 0,\exists i$
**then**7:    The subproblem *t* is valid and add it into VP;8:**end if**9:Compute the number of elements $N_{VP}$, in set of VP;10:for $k=1:N_{VP}$11:Solve the optimization problem defined in Equation (1), subject to constraints defined in Equations (2)–(10),12:**if**
$\sum n_{i,t}>N_{V,i},\exists i$
**then**13:    Add a new constraint: $n_{i,k}\leq N_{V,i}-\sum n_{i,t}$ as constraint (11);14:    Recompute the optimization problem defined in Equation (1), subject to constraints (2–11);15:    Delete the constraints defined in Equation (11);16:    **if**
$\sum n_{i,t}=N_{V,i},\exists i$
**then**17:        Add new constraints: $Q_{l,i,k+1}=0,\forall l$ as constraint (12);18:        **if**
$\sum n_{i,t}=N_{V,i},\forall i$
**then**19:           **break**20:        **end if**21:    **end if**22:**end if return** The optimized number of EVs $n_{i,t}$.


## 4. Performance Evaluation

In this section, we show the performance evaluation of our proposed schemes. We first present the evaluation methodology and then show the evaluation results.

### 4.1. Evaluation Methodology

**Figure 2:**
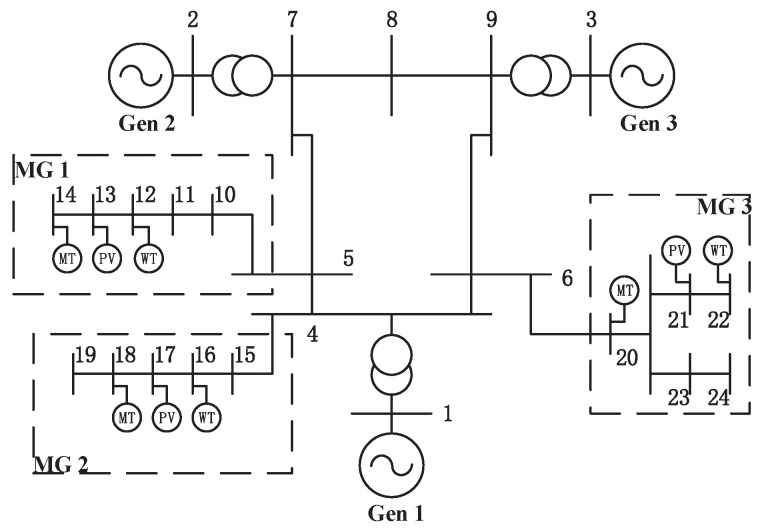
Modified 9 bus test system.

To evaluate the effectiveness of the proposed schemes, we have conducted experiments on a modified IEEE 9-bus test system, which is shown in Figure 2. In our experiments, we assume that the distance between two adjacent nodes is one. Due to the differences in geographical locations, the power losses during the power transmission among MGs are also distinct. The same generation facilities are deployed in each MG, including wind turbines, solar panels, and Diesel Generators (DG). Because of the different load demands in MGs, the capacities of three diesel generators are different. The parameters of the emission and operation cost models of diesel generators are listed in Table 2 and Table 3, respectively. Generally speaking, 1 kg diesel fuel consumptions of the DG will produce 2.708 kg CO${}_{2}$, and the emission cost created by CO${}_{2}$ is estimated as 0.014 $/kg [17,18]. If the DG consumes 1 ton diesel fuel for power generation, the emission cost will be $37.912 as a result. In addition, the maximum power output of DGs is set to satisfy the load demand in each MG, combining with the amount of generation from RESs. In this paper, the integer and binary Particle Swarm Optimization (PSO) [6] is used to solve the SOP and DOP problems.

Because of the size difference of MGs, the maximum capacities of vehicles are different. According to Equation (7), there are approximately 100, 150, and 200 vehicles existing in $MG_{1}$, $MG_{2}$ and $MG_{3}$. Based on the study in [6,16], it can be estimated that the average distance that each vehicle drives will be 32.88 miles per day and 17.89 kg CO${}_{2}$ will be produced. Thus, the emission cost of a CV is $0.25. The capacity of a lithium battery in EV is set to 10 kW. For the sake of simplicity, it is assumed that the EV will be installed with the lithium battery ($P_{EV}^{cha}=10kW$) for charging, and the minimum level $\phi_{min}$ for driving is 50%, meaning that each EV will discharge half of the battery to the power grid in order to reduce the supply pressure. In addition, the battery degradation factor $\lambda_{1}$ is 0.03 and the power loss factor $\lambda_{2}$ is 0.027, respectively. The line resistance of unit distance *R* is set to 0.2Ω, and the transmission voltage *V* between two MGs is 35 kV. Finally, the entire scheduling period is divided into 24 intervals per day. The features of three MGs in our system are listed as follows: $MG_{1}$ is with a lower load demand while with sufficient RESs, $MG_{2}$, is with a medium load demand level while with fewer RESs, and $MG_{3}$ is with the largest load demand while with the least amount of RES integration.

**Figure 3:**
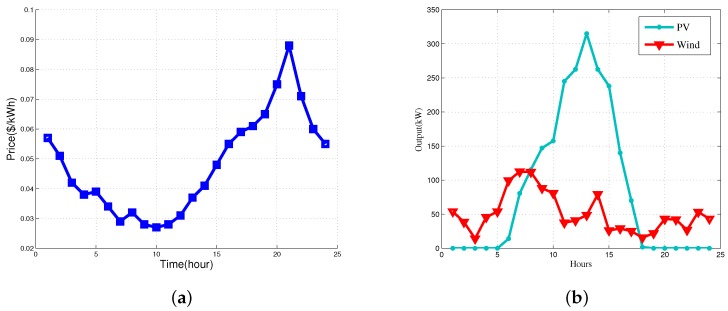
Prediction results of (**a**) real-time prices; (**b**) solar and wind.

In this paper, we consider the parameters to capture uncertainties, wind, PV generation capacity and electricity prices. Notice that wind output power and solar outputs can be derived based on the wind speed, solar irradiance, and ambient temperature. Our prior work in [12] showed that the machine learning-based schemes (e.g., Support Vector Machine (SVM), *etc.*) could be used to predict the wind speed. These learning-based schemes can also be used to predict the electricity price. In addition, in our prior work in [19], we proposed a Kalman filter-based scheme to forecast sunshine hours and ambient temperature accurately, which are associated with PV generation. For the sake of simplicity, in this paper, we assume that the parameters associated with uncertainties are distributed independently. Figure 3a shows the prediction results of real time price in the archipelago microgrid. It can be seen that the electricity prices are low in 04:00–14:00, so that the consumers are more willing to charge their EVs at these time slots. During the time slots with high prices (16:00–24:00), a smaller number of EVs will be employed to charge. Figure 3b indicates the forecasted results of wind and solar generation, where the green and red curves are the generation output of solar and wind energy in 24 h a day, respectively.

Based on the distributions of uncertainty parameters in the SOP model, we have used the Monte Carlo-based method [20] to generate 1000 scenarios for each uncertainty parameter, and the probability of each scenario is 1/1000. Each scenario contains the hourly load, real-time price, and wind and PV generated capacity. Notice that, in practice, a large number of scenarios can result in the increase of computation time and complexity, while a small number of scenarios generated by the Monte Carlo-based method can lead to the decline of accuracy. To balance the tradeoff between computation time and accuracy, we have used the fast-forward scenario reduction mechanism [21] to reduce 1000 scenarios to 10 ones.

### 4.2. Evaluation Results

#### 4.2.1. Results of SOP and DOP

**Figure 4:**
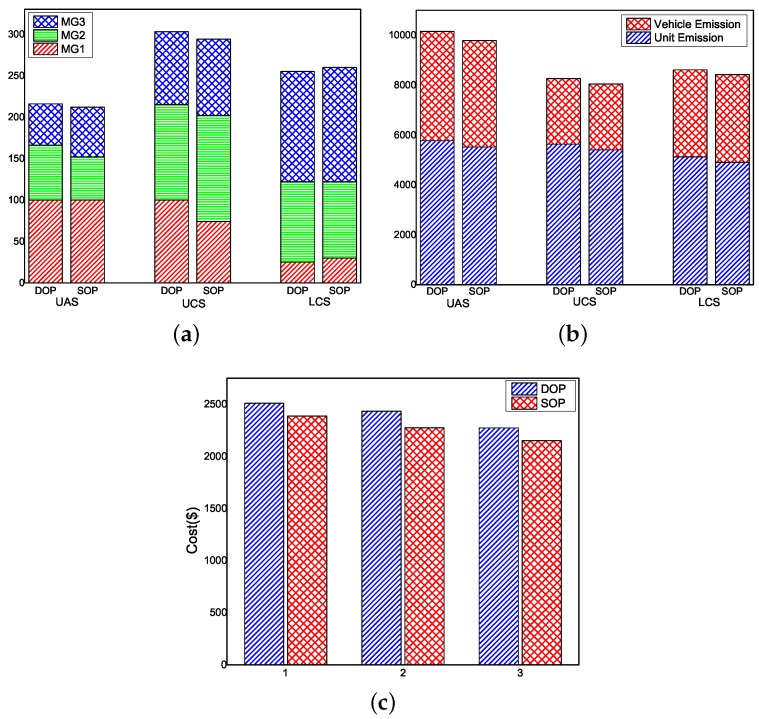
Results of SOP and DOP: (**a**) EV scales; (**b**) emissions; and (**c**) total cost in three schemes.

The different scheduling results of EV scale, by adopting three schemes in both the SOP and DOP models, are shown in Figure 4a. Recall that the surplus energy in MGs is utilized for EV charging to reduce the emission cost. It is worth noting that, although a large number of EVs are able to promote the energy efficiency of RESs and reduce the emissions cost, more EVs also signify a higher operation cost because of the frequent battery degradation and power transmission loss. To this end, it is critical to penetrate the rational number of EVs according to the three schemes of UAS, UCS and LCS.

In particular, as we can see from the figure, the total number of EVs for three schemes are 212, 294 and 260 in the SOP model, while 205, 303 and 255 in the DOP model, respectively. As we mentioned before, the UAS is a scheme that allocates EVs for charging. Apparently, we can observe that, from the results of the SOP model, in $MG_{1}$, due to sufficient RESs being integrated, 100 EVs (equal to the upper limit of the number of EVs in $MG_{1}$) are allowed to be charged. Nonetheless, in $MG_{2}$ and $MG_{3}$, because of less RESs and higher load, only a small number of EVs are allowed to be deployed. Without the power transmission among MGs, an amount of unused RES power in $MG_{1}$ is wasted. As a consequence, the UAS directly results in the smallest scale of EVs, and only 212 EVs can be deployed in the system. In the UCS, because the transmission of surplus energy is allowed among local MGs, the remaining power in $MG_{1}$ can be transported to other MGs in order to charge more EVs and offset the load. Thus, the scales of EVs in $MG_{2}$ and $MG_{3}$ are increased to 294. In addition, in the LCS, due to the peak load limitation and tariff compensation, the total number of EVs is reduced consequently. The EV scale in $MG_{1}$ is sharply reduced from 100 to 30. On the other hand, due to the higher load capacity, $MG_{2}$ and $MG_{3}$ are slightly affected. The same trend can be observed from the results of the DOP model. Notice that, although similar EV penetration scales are obtained from both the SOP and DOP models, their emissions and operation cost are totally different.

Figure 4b illustrates the total emissions caused by local units and CVs in three schemes based on the SOP and DOP models, respectively. Regarding the SOP and DOP models, the total emissions in the SOP model are reduced by 3.66%, 2.7% and 2.3%, respectively, in comparison with that of the DOP model in three scheduling schemes. We can observe that the emissions from local units are reduced in the SOP model, which is due to the fact that, with the consideration of the uncertain parameters related to RES generations and electricity prices, the results are closer to reality. Thus, less reverses are required to compensate the forecasting errors of RES generation. In addition, an accurate prediction of electricity results in the rationally charging behavior of consumers, leading to an avoidance of triggering new peak loads for the system. As a consequence, the emission caused by the local units is reduced.

Regarding the three schemes, the CV emission is highly related to the number of CVs. Thus, in the UAS, the system emission caused by CVs is the largest. In the UCS, because of maximum integration of EVs, the emission from vehicles is less than that of others. Particularly, with the transmission among local MGs and local generation for EV charging, the unit emissions in the UAS and UCS are nearly equal. Meanwhile, the penetration of EVs has greatly improved in the LCS. In the LCS, due to the shedding of the number of EVs, the generation from the local unit also declines. Nonetheless, more CVs lead to a higher total emission than the UCS. In comparison with the UAS, 15.2% reduction of emissions is realized by utilizing the LCS.

Figure 4c illustrates the total costs of three schemes in the SOP and DOP models. From the figure, after considering the uncertainties of RES generation in the SOP model, the local units’ emissions will be reduced, leading to the reduction of operation cost. In addition, by addressing the uncertainty raised by electricity price, the users may make rational decisions on EV charging and discharging. This will result in a lower tariff compensation and operation cost. Specifically, taking the results in the SOP model as an example, in the three schemes, we can observe that the maximum cost occurs in the UAS, because a lower energy efficiency of RES directly leads to a higher emission cost. In addition, in the UCS, because of the significant increase of EV scale, the corresponding operation cost increases as well. In comparison with the UAS, the operation cost saving is 4.8%. In the LCS, with the consideration of peak load limitation, the EV scale is narrowed and the total cost is reduced as well. This is because, without shaping new load peaks, fewer local unit emissions and higher energy efficiency can be realized. A great performance on cost reduction can be realized when the LCS is used (e.g., approaching 11.2% of savings over UAS). As a result, we can conclude that the LCS achieves the best performance among the three schedule schemes.

#### 4.2.2. Sensitivity Analysis

As the LCS is the most realistic and reasonable scheduling scheme in this paper, we have conducted a sensitivity study of key parameters, including weighting factor, compensation factor, renewable energy resources fluctuations, and peak load limits in the SOP model. The detailed results are shown below.

**Figure 5:**
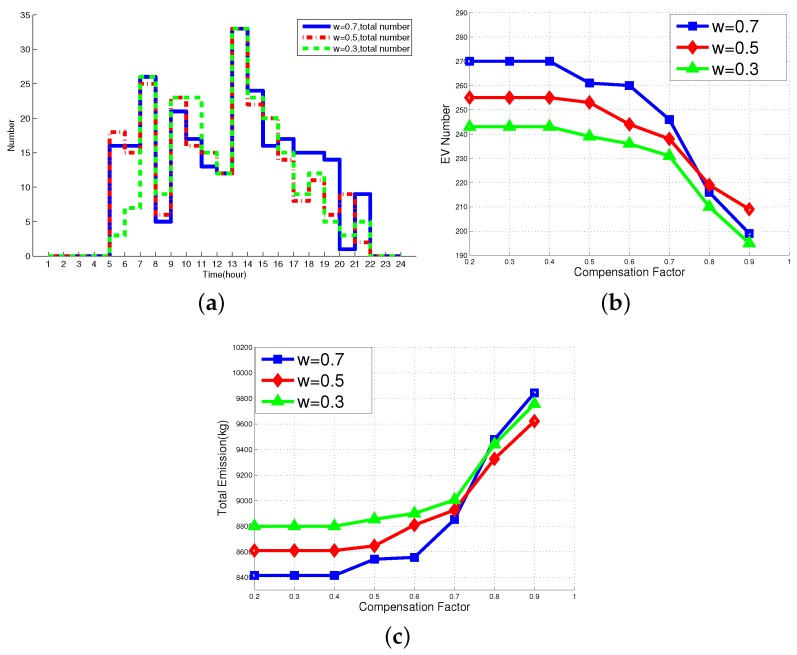
Sensitivity analysis results of weighting factor and compensation factor. (**a**) EV scale *vs.* weighting factor; (**b**) EV scales *vs.* compensation factor; (**c**) emissions *vs.* compensation factor.

Weighting factors *vs.* the Number of EVs: Figure 5a shows the total number of EVs within three MGs at each time slot versus various weighting factors. As we can see from the figure, when ω=0.5, the emission cost and the operation cost are equal so that the scheduling results will not favor to emission and operation cost. In particular, if ω>0.5, the environmental benefit is more important than the economic benefit. Obviously, the tariff compensation will have less impact on the distribution of EVs. In addition, if ω<0.5, the weight of operation cost is higher so that the tariff compensation will play a more essential role in the scheduling process of EVs, and the level of electricity prices will affect the number of EVs directly.

When ω=0.7, the distribution of EVs has less sensitivity to the price. During the time slots of 19:00–22:00 with high prices, 21 EVs are employed for charging regardless of the tariff compensation caused by a higher price gap. When the value of *ω* is reduced to 0.3, the effect of the tariff compensation increases obviously. For example, in the time slot of 19:00–22:00, there are only 13 EVs left due to the high compensation. More EVs are expected to charge at low price periods (09:00–11:00). In the case of ω=0.5, 17 EVs are arranged to charge at the same time slots. To this end, we can adjust the number of EVs by changing the weighting factors easily in order to realize the different goals between environmental and economic benefits.

Compensation factor *vs.* the number of EVs: The variation of the number of EVs *versus* different compensation factors *μ* is showed in Figure 5b. As we can see from the figure, at first, with the increase of *ω*, the proportion of emissions cost in the objective function grows. More EVs should be deployed to reduce the emissions created by those CVs. At the same time, due to the limit of the quantity of the RESs and the peak load constraint, the raise of *μ* will lead to a shedding on the optimized EV scales. As shown in the figure, when *μ* is smaller, the number of EVs has a slight reduction even no change from the initial number. Nonetheless, when μ≥0.5, the number of EVs begins to decline and the number of EVs declines faster in ω=0.7 than that of ω=0.5,0.3. It can be explained that the larger of the *ω*, the more EVs are used for charging during the time slots with high prices. Thus, the additional EVs can be easily affected by the increase of the tariff compensation and the curve becomes steeper when ω=0.7.

**Figure 6:**
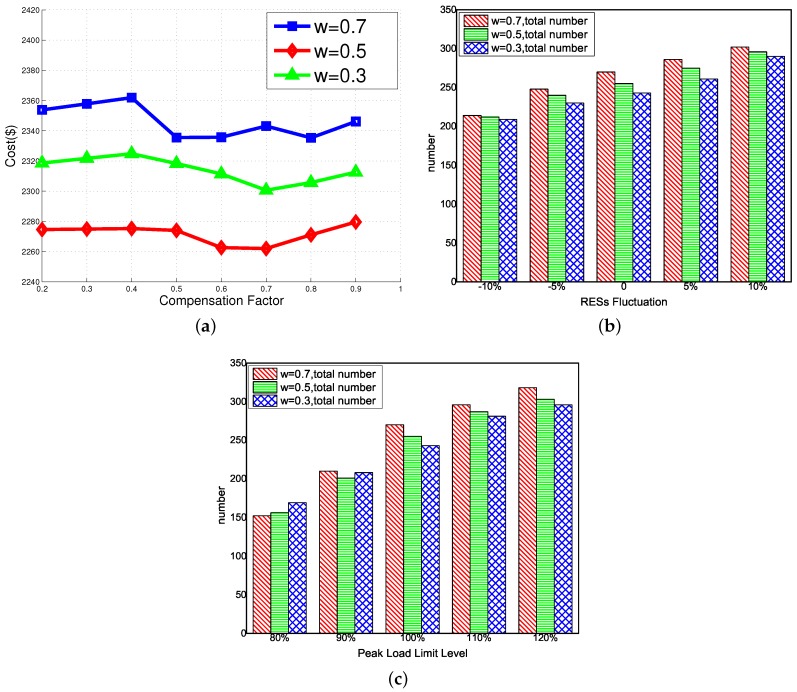
Sensitivity analysis results of compensation factor, RES fluctuations and peak load limit. (**a**) operation cost *vs.* compensation factor; (**b**) RES Fluctuation *vs.* the number of EVs; (**c**) peak load limit *vs.* the number of EVs.

Compensation factor *vs.* emissions and total cost: Figure 5c and Figure 6a illustrate the change of emissions and the total cost with the increase of the compensation factor *μ*, respectively. As shown in Figure 5c, we can observe that the reduction of the number of EVs leads to a great shedding on CO${}_{2}$ emission although the local units emission has a slight promotion. Thus, the overall emissions trend is upward. The total cost is shown in Figure 6a. At first, the small value of *μ* has no impact on the number of EVs so that the total cost increases a little because of the promotion of tariff compensation. In addition, when *μ* varies from 0.4 to 0.7, the results of the tariff compensation improvement cannot compensate the battery degradation cost and power loss penalty on the total cost. When μ≥0.8, a larger shedding of the EV scale and the higher tariff compensation play a more essential role on the total cost. As a result, the cost curve rises when *μ* is larger.

RES fluctuations *vs.* the number of EVs: As an essential clean energy resource in MGs, RESs play a significant role in power supply and are environmentally friendly. Nonetheless, due to the intermittency and uncertainty, it is difficult to make an accurate prediction of the amount of the power generation made by RESs. This will affect the reliability and stability of MGs. In our proposed archipelago microgrid system, the fluctuation of RESs will directly affect the generation of local units, leading to the diversification of the number of EVs. Moreover, recall that if more local unit generations are used to charge EVs, more CO${}_{2}$ emissions will be generated than the emission caused by CVs. Thus, the quantity of RESs is highly correlated to the number of EVs. As shown in Figure 6b, we investigate the variation of the number of EVs with different prediction errors of RESs (from −10%–+10%). It can be seen that a decreasing scale is illustrated with the reduction of RES generation. When there are 90 % of predicted RESs, only 214, 212 and 209 EVs can be deployed in each MG, while 302, 296 and 290 EVs can be deployed with +10% more RES generation while considering the limit of peak load, showing a great improvement of the number of EVs.

Peak load limits *vs.* the number of EVs: As we mentioned above, the peak load limit is another important constraint to ensure the stable operation of the power grid system. We investigate the impact of the level of peak load limits on the number of EVs. Results are shown in Figure 6c. From the figure, we can see that when the peak load limit level is less than 100%, the scales of EV are reduced in each MGs with the shedding of peak load capacities. Recall that the higher the value, the more EVs can be deployed at slots when high prices occur since there is less tariff compensation. Meanwhile, the electricity price is high correlate to the load level. In comparison with the case where ω=0.3,0.5, when ω=0.7, the most EVs will be deployed at the time slots when a high load demand occurs, the number of EVs can be easily affected by the reduction of peak load capacity. Thus, the number of EVs declines fastest when ω=0.7. In addition, with the increase of peak load capacity, more EVs can be deployed in the system. In particular, when the level of peak load limit rises to 120%, the SOP model with the LCS is equivalent to the one with UCS, the number of EVs in each MG reaches to the optimal value of 318, 303 and 296, respectively.

## 5. Related Works

In recent years, the IMG has attracted much attention in the research community [22,23,24,25,26,27,28,29,30,31,32,33]. For example, Kahrobaeian *et al.* in [25] proposed a hybrid distributed network-based power control scheme to enhance the dynamic characteristics of MGs, minimize the power-sharing error, and improve the system stability for islanded MG that consists of the distributed power generators. An objective of minimizing the fuel cost was used in [26] to ensure the stable MG operation in an islanded model with two-sharing principles: fixed droop and adjustable droop. Alipour *et al.* in [27] presented a stochastic programming framework for distributed energy resources, the Combined Heat and Power (CHP) system, and energy storage devices, with an objective of realizing the profit maximization. In their study, three cases with the islanded model, the grid-connected model, and the proposed demand response program were investigated.

There are several research efforts in which the MG models are similar to our proposed archipelago microgrid. For example, Rua *et al.* in [24] presented a hierarchical control scheme to evaluate the uncertainties in an isolated multi-microgrid system when the data exchange occurred in communication facilities. Pereira *et al.* in [28] conducted the analysis of the communication in an islanded multi-microgrid. The packet delivering delay and the loss of communications were considered to assess the performance of the hierarchic structures. Nonetheless, within the aforementioned proposed systems, the scheduling of EVs was not considered.

As an essential component of MG, EV has attracted a growing attention in the research community [3,5,6,34,35,36,37,38,39,40,41,42]. For example, the optimized operation of EVs in islanded MG were considered in [3,5]. Abdelaziz *et al.* in [3] proposed a new multistage algorithm to minimize the reduction of loads and the operation cost of MGs and meet the demand of consumers for EVs by considering the uncertainty of RES generation and EV charging. An objective of minimizing the operation cost and maximizing the profit of IMG was studied by scheduling the EV charging and battery swapping station with a fuzzy control approach in [5]. In [35], a non-cooperative game problem was formulated to achieve multiple goals, including the reduction of the cost associated with users, improving the social benefit, and enhancing the stability of the MG by treating the EV as a storage device. Gan *et al.* in [36] developed a decentralized algorithm to seek a rational charging plan for EVs to fulfill the power valley in power grids. Although there have been a number of research efforts on EV charging scheduling, not many research efforts on the scale of EVs have been carried out. Saber *et al.* in [6] deployed the EVs to simply replace all the CVs in the MG, without considering the great increase in the investment costs and the consumers’ preferences for CVs.

The main difference between our investigation and other existing research efforts is that we have proposed both stochastic and deterministic optimized models for minimizing the total emission and operation cost in the novel archipelago microgrid. Our proposed system can enable the aggregator to deploy a reasonable number of EVs with a consideration of the restriction of generation capacity and the reliability and stability of the system. The evaluation data shows that a desired improvement of our proposed schemes are energy efficiency, environmental friendliness, and cost reduction.

## 6. Conclusions

In this paper, we have addressed the optimal EV penetration issue in a novel “archipelago microgrid” system. In such a system, the power transmission among local MGs are allowed to promote energy efficiency and assist with a larger amount of EV penetration. The optimal EV penetration problem has been formalized as the two-stage Stochastic Optimal Penetration (SOP) model, in which the uncertain parameters are captured by the Monte Carlo-based scheme, aiming to minimize the emission and operation costs. For the sake of comparison, the Deterministic Optimal Penetration (DOP) model is proposed as a baseline scheme that originally appeared in a much shorter conference version [9]. To rationally utilize the surplus energy and deploy the reasonable scale of EVs in the system, we have proposed two scheduling schemes: UCS and LCS. Our extensive experiments on a modified IEEE 9-bus system demonstrate that the SOP model is able to realize a significant reduction in the environmental pollution raised by CO${}_{2}$ emission and the operation costs in the system than the DOP model. With respect to the UCS and LCS, the LCS can achieve more realistic performance than the UCS, while achieving an effective reduction of emission and operation costs. In addition, a sensitivity analysis has been conducted to evaluate the efficiency of the LCS in the SOP model.

## Figures and Tables

**Table 1:** Notations.

NG,NM,NT,NS:	The number of local units, MGs, time slots, and scenarios
$\phi_{min}$:	The minimum battery energy stored for handling EV’s normal driving activities
*ω*:	Weighting factor
*μ*:	Compensation factor of price gaps
$\lambda_{1},\lambda_{2}$:	Penalty factor of battery capacity degradation and power losses
$\eta_{cha},\eta_{dis}$:	Charging/discharging efficiency of storage battery
$a_{i,j},b_{i,j}$:	Fuel consumption coefficients of DG *j* in MG *i*
$\alpha_{i,j},\beta_{i,j}$:	The operation cost coefficients of DG *j* in MG *i*
$E(P_{i,r,t})$:	Cost of unit *r* to compensate RES prediction errors in MG *i* at time *t*
$n_{i,t}^{cha},n_{i,t}^{dis}$:	The number of EVs charged/discharged at time *t* in MG *i*
$r_{min},r_{it}$:	Minimum and real-time electricity price during the day ($/kWh)
$R_{i,l}$:	Line resistance between MG *i* and *l* (*Ω*)
*V*:	Transmission voltage among MGs (kV)
$Q_{i,l}$:	Transported power between MG *i* and *l* (kW)
$N_{EV,i},N_{CV,i},N_{V,i}$:	The number of EVs, CVs and total vehicles in MG *i*
$P_{loss}$:	The power losses during power transmission (kW)
$P_{i,j,t}$:	Power generation of local unit *j* (kW)
$P_{load,i,t}$:	Non-EV load in MG *i* at time *t* (kW)
$P_{Pv,i,t},P_{Wind,i,t}$:	Power generation of PV and wind (kW)
$SU_{i,j},SD_{i,j}$:	Startup and shutdown cost of unit *j* ($)
$Ru_{i,j},Rd_{i,j}$:	Ramp-up/down limit of unit *j* (kW)
$P_{EV,i,t,k}^{cha},P_{EV,i,t,k}^{dis}$:	Charging/discharging power of the $k^{th}$ EV (kWh)
$P_{i,j}^{min},P_{i,j}^{max}$:	Minimum/maximum power generation of unit *j* (kW) in MG *i*
$P_{EV}^{max},P_{EV}^{min}$:	The maximum and minimum capacity of EV’s battery (kWh)
$I_{i,t},U_{i,t}$:	Charging/discharging status of EV, where 0 indicates charging and 1 indicates discharging, respectively
$x_{i,j,t}$:	Operation status of unit *j*, where 0 means the unit’s stop status and 1 indicates its operation status, respectively
$y_{i,j,t},z_{i,j,t}$:	Startup and shutdown status of unit *j*, where 0 and 1 means startup and shutdown, respectively
SC, IC:	Slope Coefficient and Intercept Coefficient of fuel consumption per unit generation

**Table 2:** Coefficients of fuel consumption curve.

Rated Power (RP) (kW)	SC (a,L/h)	IC (b,L/h)
30–100 kW	0.273	0.033
100–300 kW	0.253	0.028
>300 kW	0.244	0.014

**Table 3:** Operation cost index.

MG	Type	*α* ($)	*β* ($/kWh)	$P_{\min}$ (kW)	$P_{\max}$ (kW)
1	DG	15	0.13	20	200
2	DG	25	0.35	20	400
3	DG	40	0.50	20	500
**MG**	**Type**	**${\mathrm{SU}}_{i,j}$ ($)**	**${\mathrm{SD}}_{i,j}$ ($)**	**${\mathrm{Ru}}_{i,j}$ (kW)**	**${\mathrm{Rd}}_{i,j}$ (kW)**
1	DG	50	5	30	10
2	DG	30	3	40	20
3	DG	20	2	50	30
